# Socioeconomic factors and severity of periodontal disease in adults (35-44 years). A cross sectional study

**DOI:** 10.4317/jced.54033

**Published:** 2017-08-01

**Authors:** José-Manuel Almerich-Silla, Pedro J. Almiñana-Pastor, Montserrat Boronat-Catalá, Carlos Bellot-Arcís, José-María Montiel-Company

**Affiliations:** 1 Tenured lecturer, Preventive Dentistry Teaching Unit, Department of Stomatology, Faculty of Medicine and Dentistry, University of Valencia (Spain); 2 Grado en Odontología [equivalent to BDS],, Specialist Master of Periodontics, Department of Stomatology, Faculty of Medicine and Dentistry, University of Valencia (Spain); 3 PhD, Associate lecturer, Orthodontics Teaching Unit, Department of Stomatology, Faculty of Medicine and Dentistry, University of Valencia (Spain); 4 Post-doctoral contract lecturer, Preventive Dentistry Teaching Unit, Department of Stomatology, Faculty of Medicine and Dentistry, University of Valencia (Spain)

## Abstract

**Background:**

Periodontal disease or periodontitis is an inflammatory disease with a hight prevalence. According to the last oral health survey of the Spanish population, between 24% and 37% of Spaniards aged over 35 years have periodontitis and 6% to 10% of the adult population have deep periodontal pockets. The aim of this study was to determine the association between risk factors and the presence of periodontal pockets in the adult population.

**Material and Methods:**

A cross sectional or prevalence study of a representative sample of the adult population of the Valencia region was designed. The sample was recruited at 35 health centres, The study was conducted in November and December 2006 under standardized conditions as regards light sources, equipment and instruments and the position of the three previously calibrated dentist examiners.

**Results:**

The sample examined consisted of 733 individuals (220 men and 513 women). Measured by the CPI, 13% were healthy and 5.5% presented bleeding. The prevalence of calculus was 59.3%, that of 3.5-5.5 mm pockets was 15.8% and that of pockets deeper than 5.5 mm was 4.6%. Almost half the sextants were healthy (2.89), 0.61 presented bleeding and 1.74 presented calculus. The mean number of sextants affected by 3.5-5.5 mm pockets was 0.46 and 0.07 presented deep pockets (>5.5 mm). An adjusted multiple logistic regression model with the presence of periodontal pockets as the dependent variable showed that the significant independent variables were low social class (OR=1.81), smoking (OR=1.68), primary education (OR=1.57), male gender (OR=1.56) and age (OR=1.08). The other study variables were not significant in this model.

**Conclusions:**

Socioeconomic factors such as primary education and low social class, as well as gender, age and smoking, were found to be associated to a significant degree with greater prevalence of periodontal disease in the adult population.

** Key words:**Periodontal disease, adults, socioeconomic factors, periodontal pockets, cross sectional study.

## Introduction

Periodontal disease or periodontitis is an inflammatory disease of the tissues that support the teeth, caused by specific microorganisms or groups of microorganisms, which results in progressive inflammatory destruction of the periodontal ligament and the alveolar bone and the formation of periodontal pockets, or receding gums, or both. The clinical sign that distinguishes gingivitis from periodontitis is the loss of attachment that occurs in periodontitis (1). According to the 2015 oral health survey of the Spanish population, between 24% and 37% of Spaniards aged over 35 years have periodontitis and 6% to 10% of the adult population have deep periodontal pockets (2).

Today, the diseases that affect the periodontium continue to be classified according to the proposal made by Armitage in 1999 (3). This classification divides the different clinical entities into the following major groups: gingival diseases, chronic periodontitis, aggressive periodontitis, periodontitis as a manifestation of systemic diseases, necrotising periodontal diseases, abscesses of the periodontium, periodontitis associated with endodontic lesions, and developmental or acquired deformities and conditions (3,4).

The clinical signs used to diagnose periodontal disease are probing depth, bleeding on probing, and clinical attachment level.

Periodontal disease presents multi-factorial aetiology (5). The main risk factors for its progression are the presence of plaque, calculus and gingivitis, although many studies have shown that the these clinical factors alone are not sufficient for loss of attachment to appear (5-8).

One of the environmental factors most associated with the progression of periodontal disease is smoking. Smokers are 2 to 7 times more likely to suffer periodontitis. Heavy smokers are twice as likely to present loss of attachment and bone loss than light smokers. Diabetes is another risk factor for periodontitis and poor control of diabetes in the presence of calculus is associated with a higher frequency of periodontal pockets ≥ 4 mm (9,10).

Other factors related to periodontal disease are genetic factors, ethnicity, advanced age, male gender, depression, traumatic occlusion, osteoporosis, the presence of red complex bacteria (Porphyromona Gingivalis, Tanarella Forsythus, Treponema Denticola) and Prevotella Intermedia, low educational level and lack of dental care. In the presence of heavy accumulations of calculus, they are associated with a loss of periodontal support and may be considered indicators of a risk of periodontitis (11).

The aim of this study was to measure the association between different risk factors and the presence of periodontal pockets in the adult population.

## Material and Methods

A cross sectional or prevalence study of a representative sample of the adult population of the Valencia region was designed. Out of the 111 health centres in the Valencia Region of Spain, 35 were selected at random. At these health centres, persons aged between 35 and 44 years who visited the health centre for a non-dental reason were asked whether they would agree to being examined. The acceptance level was 77%.

Data collection was carried out in November and December 2006 under standardized conditions as regards the light source, equipment and instruments and the position of the three dentist examiners and the three recorders, who were all previously calibrated over several sessions and obtained a weighted kappa score of >0.8 compared to the gold standard.

The examination was performed with the person to be examined sitting on a chair, with his or her neck extended, opposite the examiner. The lighting was constant during the examination process, as a portable white-spectrum lamp was used.

The variables recorded in this study were.

- Community periodontal index (CPI), according to the WHO recommendations, based on 6 teeth (17/16, 11, 26/27, 36/37, 31, 46/47) classed as 0=healthy, 1=bleeding, 2=calculus, 3=3.5-4.4 mm pocket, or 4= >5.5 mm pocket.

- Education (primary, secondary or higher).

- Social class, following the Spanish Society of Epidemiology’s classification into low, middle or high class based on occupation.

- Immigrant (yes/no)

- Gender (male/female)

- Age

- Smoker (yes/no)

- Alcohol consumption daily (yes/no)

- Tooth brushing daily (yes/no)

- Regular visits to the dentist at least once a year (yes/no)

- Place of residence (urban/rural).

The sample size calculation was based on the 2005 Spanish epidemiological study estimate of a 25.4% prevalence of periodontal pockets. For a 95% confidence level and 3.2% precision, the estimated sample size was calculated as 722 persons.

Bivariate statistics were calculated with means, proportions and 95% confidence intervals. Student’s T-test was used to compare the means once normal distribution and the proportions had been verified by the Shapiro-Wilk test and the chi-squared test, respectively. Multivariate data analysis was also performed using forward Wald stepwise logistic regression. The significance level was set at *p*<0.05.

## Results

The sample examined consisted of 733 individuals (220 men and 513 women).

Measured by their maximum CPI score, 13% were healthy, with no sign of periodontal disorders, and 5.5% presented bleeding. The prevalence of calculus was 59.3%, that of 3.5-5.5 mm pockets was 15.8% and that of pockets deeper than 5.5 mm was 4.6%.

As regards the extent of the disease process, almost half the sextants were healthy (2.89), 0.61 presented bleeding and 1.74 presented calculus. The mean number of sextants with 3.5-5.5 mm pockets was 0.46 and 0.07 of sextants had deep pockets (>5.5 mm). The sextants excluded from the CPI calculation numbered 0.23.

Significant differences in the mean number of sextants with 3.5-5.5 millimetre periodontal pockets were found. They were higher in individuals with a primary education, low social class, male gender, smokers, and who visited the dentist regularly. As regards deep periodontal pockets (>5.5 mm), the only variable associated with a higher mean number of sextants was low social class ( Table 1).

**Table 1:**
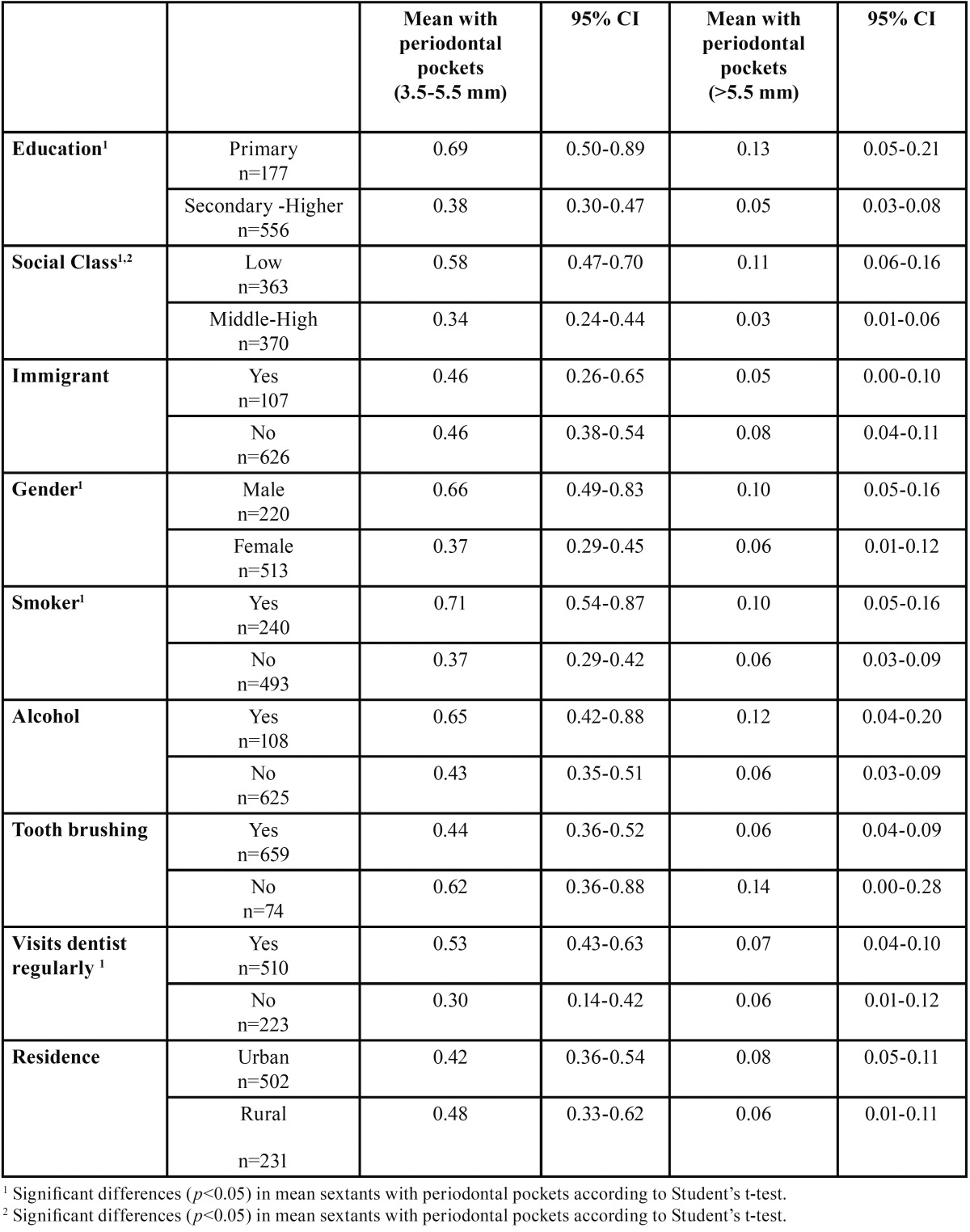
Number of sextants with periodontal pockets, by variables, in a 35-44 year-old population (n=733).

On applying a chi-squared test (*p*<0.05), an association with a higher prevalence of periodontal pockets was found for primary education, low social class, male gender, smokers, alcohol consumption, no daily tooth brushing and regular visits to the dentist ( Table 2).

**Table 2:**
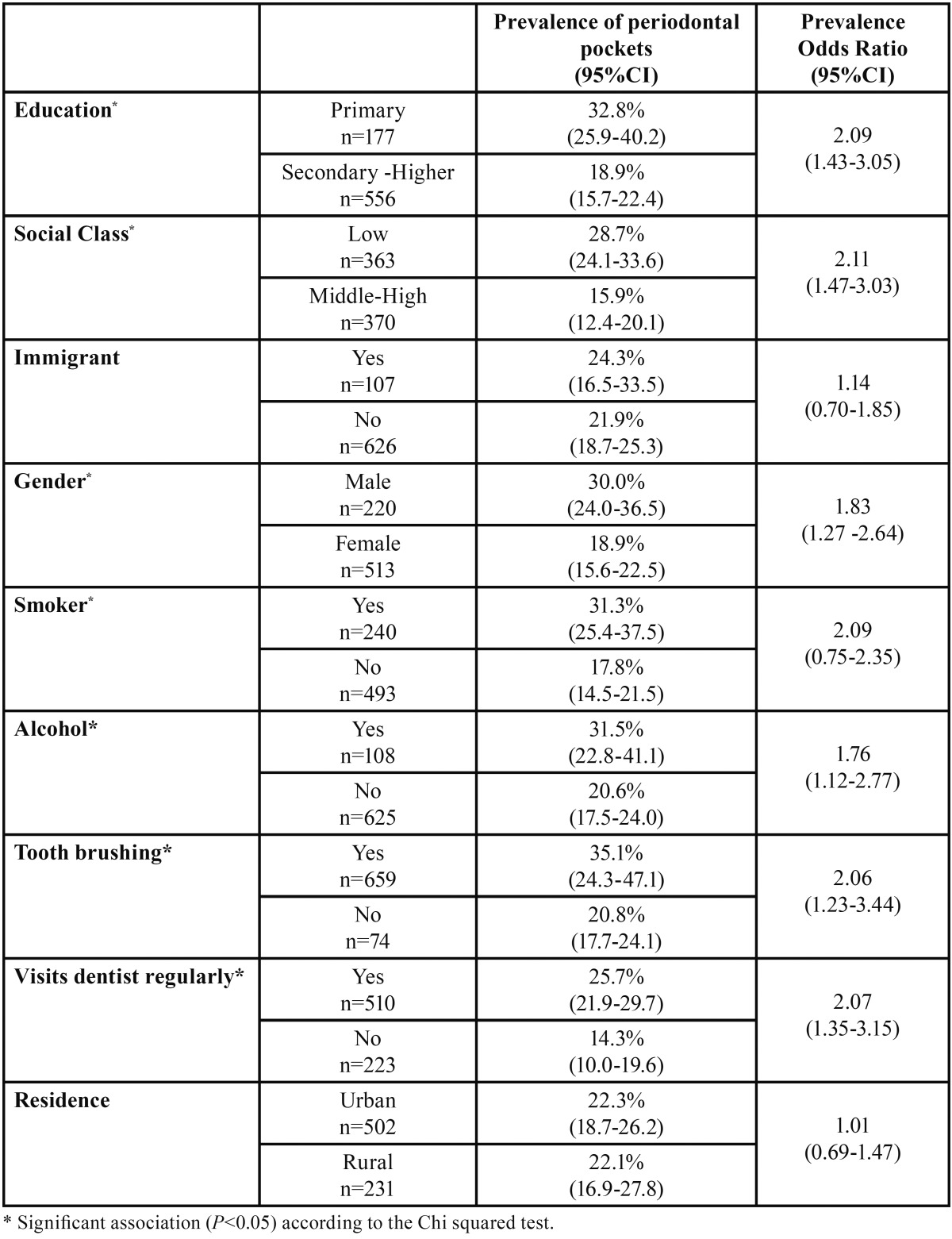
Factors associated with periodontal pockets in a 35-44 year-old population.

An adjusted multiple logistic regression model (Hosmer and Lemeshow’s test of goodness fit *p*=0.295; % correctly predic-ted=77.4%), taking the presence of periodontal pockets as the dependent variable, found that the significant independent variables were low social class, smoking, primary education, male gender and age ( Table 3). The other study variables were not significant in this model.  Table 3 shows the odds ratios (OR), which range between 1.81 and 1.08, and their respective 95% confidence intervals (95% CI).

**Table 3:**
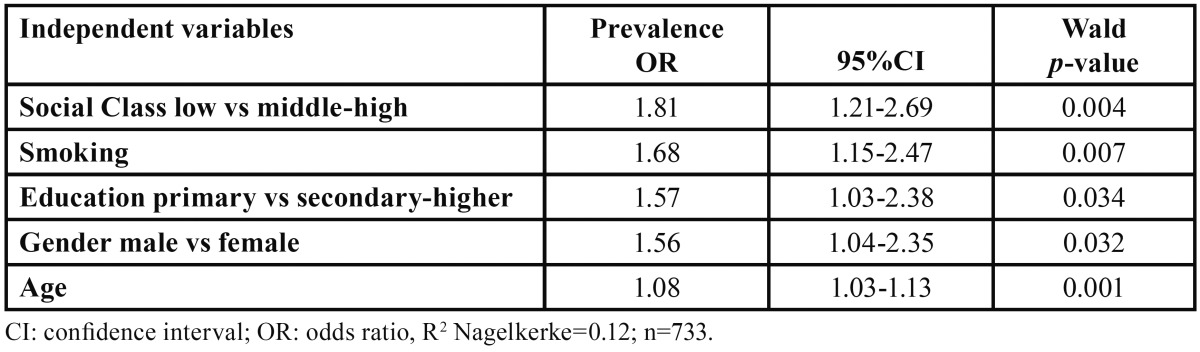
Multiple logistic regression model with periodontal disease as the dependent variable in a 35-44 year-old population.

## Discussion

In this cross sectional study of 733 individuals, the proportion of healthy individuals with no sign of periodontal disorders was 13%, the prevalence of 3.5 to 5.5 mm pockets was 15.8% and that of pockets deeper than 5.5 mm was 4.6%. On comparing these findings with similar studies, 46% of US adults had periodontitis but only 8.9% of those affected presented an advanced stage of the disease (12), while a study in a Danish population (13) found low percentages of periodontally-healthy individuals in the 35-44 year-old and 65-74 year-old groups (7.7% and 2.4%, respectively).

In Spain, studies of representative population groups of 35-44 year-olds have found the following proportions of completely healthy individuals: 19.3% in Llodrá *et al.* (14), 14.8% in Bravo *et al.* (15), 16% in Llodrá 2012 (16) and 6% in Carasol *et al.* (17), although the latter only studied the working population rather than the general population. In the present study of a representative sample of this age group in the Valencia region, the percentage was 13%. As regards the presence of a moderate level of periodontal disease, the CPI 3 results were 21%, 21.5%, 11% and 29.2%, respectively, in the above-mentioned studies, and 15.8% in the present study. Lastly, the above studies found advanced periodontal disease in 4.2%, 3.9%, 5 % and 8.4% of their samples, respectively, while the proportion of CPI 4 scores in the present study was 4.6%. These results for the Spanish population also agree with data obtained for the European population (18-20).

Another salient finding is the extent of periodontal disease. On examining the data by sextant, almost half of the sextants were healthy (2.89) and even though 1.74 presented calculus, only 0.46 sextants per individual had periodontal pockets measuring 3.5 to 5.5 mm and only 0.07 had pockets of over 5.5 mm. These findings are similar to those of other studies (6-8,21).

The present study found that low social class, age, smoking, male gender and primary education were independent variables that showed a significant association with periodontal pockets, while others such as tooth brushing or alcohol were not significant variables for periodontal disease.

Age proved to be a variable that was significantly associated with periodontal conditions, as in other epidemiological studies (12). Carasol *et al.* (17) associated age with periodontal disease with an OR of 1.43. The OR associated with age in the present study was 1.08.

Male gender was also related to periodontal disease in this study (OR=1.84), in agreement (12,22,23) or to a slightly lower extent than the association found by other authors (OR=2.15) in Carasol *et al.* (17).

On analysing the socio-economic variables, a statistically significant relationship was found between low educational level, low social class and a higher prevalence of periodontal pockets. This agrees with the findings of other studies (12,13,17,21,24-26) confirming the consistency of this association.

With regard to smoking, the OR was 1.68. Philstrom *et al.* (9) observed that among periodontal patients, smokers were five times more likely than non-smokers to present deep pockets (OR 5.3). In the present study, the sample represented the general population, not only periodontal patients, and the results confirm the great importance of smoking as a factor associated with periodontal pockets, since smokers were twice as likely to present them. Other more recent studies (17,24,27) have also reasserted the relationship between smoking and more severe periodontal disease.

Regular visits to the dentist are a factor that is related significantly to the presence of periodontal pockets, as those who visited the dentist regularly presented higher percentages of pockets (25.7 % compared to 14.3%). The relationship between visiting the dentist regularly and a higher percentage of periodontal pockets is understandable, since these could be one of the main reasons for making such visits in the first place. In a young population group, Krustrup *et al.* (13) also observed a greater prevalence of periodontal pockets among patients who visited the dentist regularly.

An aspect of the present study that should be borne in mind is the large and representative size of the sample, which makes it possible to extrapolate its findings to the adult population of the Valencia region. As regards its limitations, one is the absence of radiographic records which would show the bone loss resulting from periodontal disease, although these are not indicated in epidemiological studies and the main variable is therefore periodontal pockets. Another is that the quantity of tobacco smoked was not recorded, as some studies (9,17) have shown that heavy smokers present greater progression of the disease than those who smoke less. Also, as is well known, periodontal disease is not uniformly distributed throughout the different teeth in the mouth. Consequently, the screening protocols normally employed in epidemiological studies, like the CPI, may underestimate the preva-lence of periodontal disease (17,28).

To conclude, it may be stated that this study of a representative sample of the population of the Valencia region of Spain shows that periodontal disease is related to a series of socio-economic factors such as low educational level and social class, as well as with male gender, age and smoking. It would therefore appear to be evident that the adult population of the Valencia region includes risk groups in need of health education, prevention and treatment.
